# Supplementary material for: SUMO Modification of OsFKBP20-1b Is Integral to Proper Pre-mRNA Splicing upon Heat Stress in Rice
Source: Int J Mol Sci. 2021 Aug 22;22(16):9049. doi: 10.3390/ijms22169049 (PMC8396655; doi:10.3390/ijms22169049)
Supplement: Supplementary file 1 [file ijms-22-09049-s001.zip › ijms-1349775-supplementary.pdf]

a

**Protein ID:**	N/A
**Defintion:**	N/A
**Length:**	185 aa

```
  1 MSETIDLTGD RCILKTVIRR AKDDATAPSD SLPIVDVHYE GTLAENGEVF
 51 DTTHEDNSVF SFEIGEGTVI KAWDIAVKTM KVGEVAKITC KPEYAYGAAG
101 SPPEIPPDAT LTFEVELIAC RPRKGSSVES VSEEKARLEE LKKQREIAAA
151 AKEEEKRKRE EAKAAAAARV QAKLEAKKGK GKKAK
```

Motifs with high probability
Motifs with low probability
Overlapping Motifs

No.	Pos.	Group	Score	No.	Pos.	Group	Score
1	K173	AARVQ **AKLE** AKKGK	0.79	4	K81	IAVKT **MKVG** EVAKI	0.63
2	K152	EIAAA **AKEE** EKRKR	0.79	5	K177	QAKLE **AKKG** KGKKA	0.62
3	K22	TVIRR **AKDD** ATAPS	0.79	6	K158	KEEEK **RKRE** EAKAA	0.44

b

ID	Position	Peptide	Score	Cutoff	P-value	Type
Unnamed	34 - 38	APSDSLPIVDVHYEGTLAE	43.415	29.92	0.075	SUMO Interaction
Unnamed	135	VESVSEEKARLEELK	3.983	3.32	0.066	Sumoylation Nonconcensus
Unnamed	152	REIAAAAKEEEKRKR	5.08	2.13	0.025	Sumoylation Concensus
Unnamed	177	VQAKLEAKKGKGKKA	4.65	3.32	0.043	Sumoylation Nonconcensus
Unnamed	178	QAKLEAKKGKGKKAK	3.504	3.32	0.046	Sumoylation Nonconcensus
Unnamed	183	AKKGKGKKAK*****	3.77	3.32	0.046	Sumoylation Nonconcensus
Unnamed	185	KGKGKKAK*******	4.321	3.32	0.046	Sumoylation Nonconcensus

Supplementary Figure S1 . Prediction of SUMOylation sites in OsFKBP20-1b.
OsFKBP20-1b protein sequences were analyzed by **(a)** SUMOplot™ analysis program and **(b)** GPS-SUMO online service

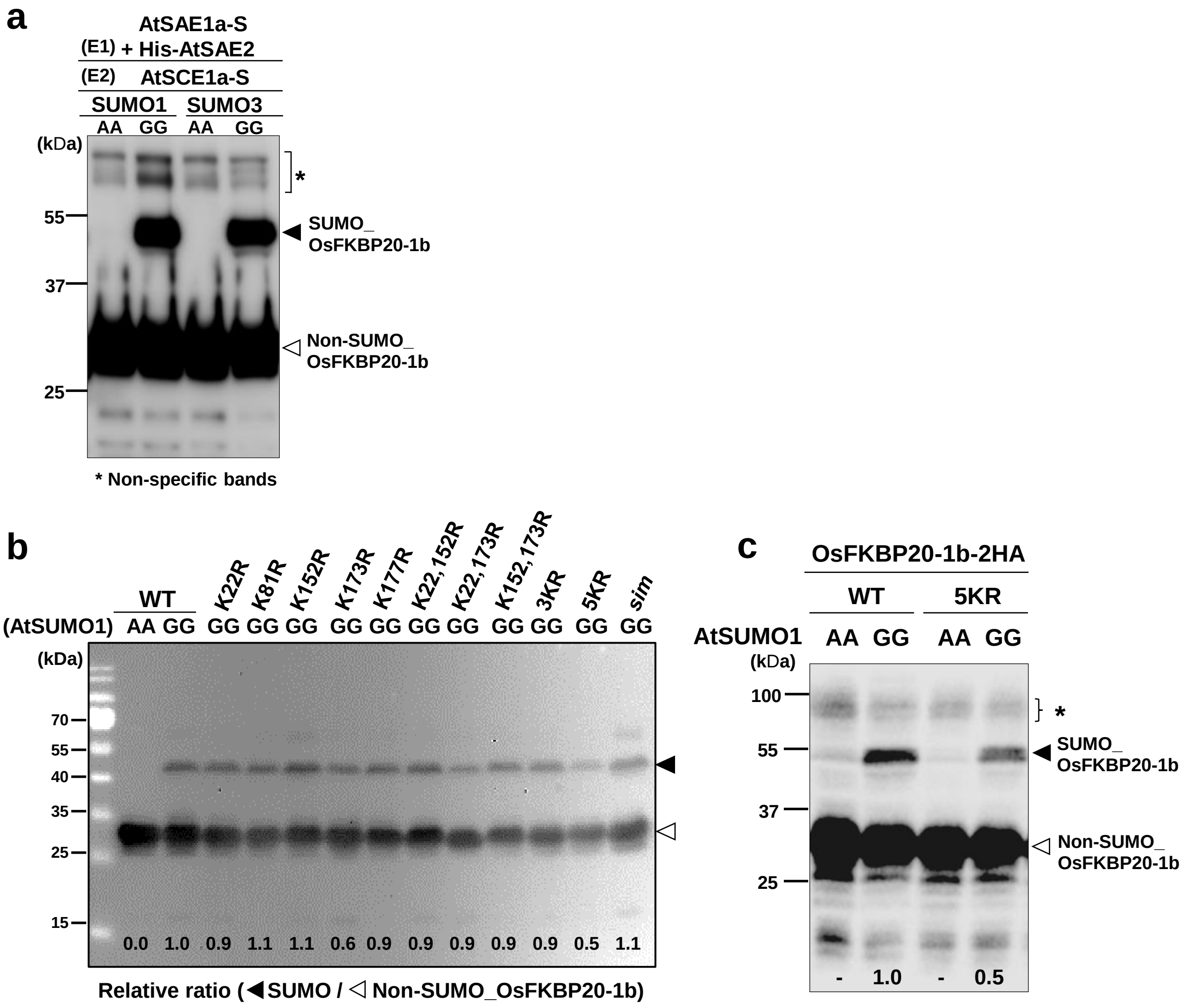

Supplementary Figure S2. *In vitro* SUMOrylation assays of OsFKBP20-1b.

a. SUMOylation assay of OsFKBP20-1b through reconstitution of the SUMO conjugation pathway in *E. coli*. HA-fused OsFKBP20-1b was expressed into the *E. coli* systems expressing the SUMOylation enzymes E1 (AtSAE1a, AtSAE2), E2 (SCE1a) and AtSUMO-1 or AtSUMO-3. After IPTG incubation, OsFKBP20-1b was detected by immunoblotting with anti-HA antibody. GG and AA stand for WT forms of SUMO and non-functional SUMO1G92,93A, respectively. b. *E. coli* cells containing in vitro SUMOylation components were transfected with OsFKBP20-1b WT or various KR mutants (3KR, K22,152,173R; 5KR, K22,81,152,173,177R, *sim,* sumo-interacting motif mutation (IVDV34-37AADA)) as indicated, and immunoblot was performed by anti-HA antibody. The relative intensities of the SUMO-modified and unmodified non-SUMO_OsFKBP20-1b were determined by a densitometry. c. *E. coli* cells containing in vitro SUMOylation components were transfected with OsFKBP20-1b WT or 5KR mutants (K22,81,152,173,177R) as indicated, and immunoblot was performed by anti-HA antibody. Asterisk means non-specific band. The relative intensities of the SUMO_OsFKBP20-1b were determined by a densitometry.

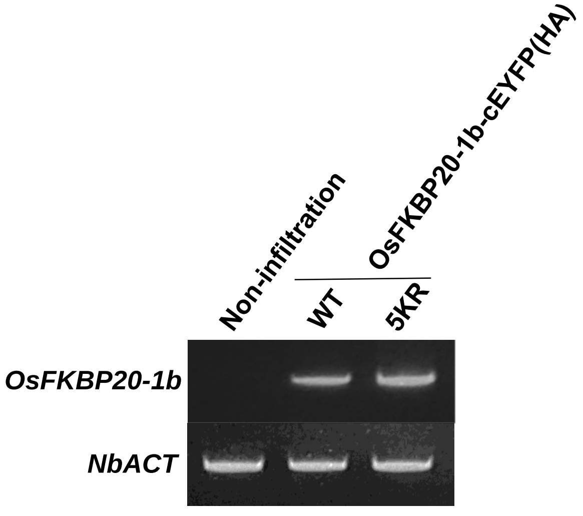

Supplementary Figure S3. Transcript levels of wild-type OsFKBP20-1b (WT) and less SUMO 5KR mutant OsFKBP20-1b (5KR) in *Nicotiana benthamia.*

OsFKBP20-1b WT and 5KR were cloned into cEYFP (HA tag) vector and co-infiltrated with GFP into the leaves of 4-week-old *Nicotiana benthamiana* and 2 days after infiltration total mRNA was extracted from the leaves. Transcription levels of *OsFKBP20-1b* in leaves of *Nicotiana benthamiana* expressing by agro-infiltration mediated transient expression. The expression levels were showed using semi-quantitative RT-PCR analysis. *NbACT* was used for loading control.

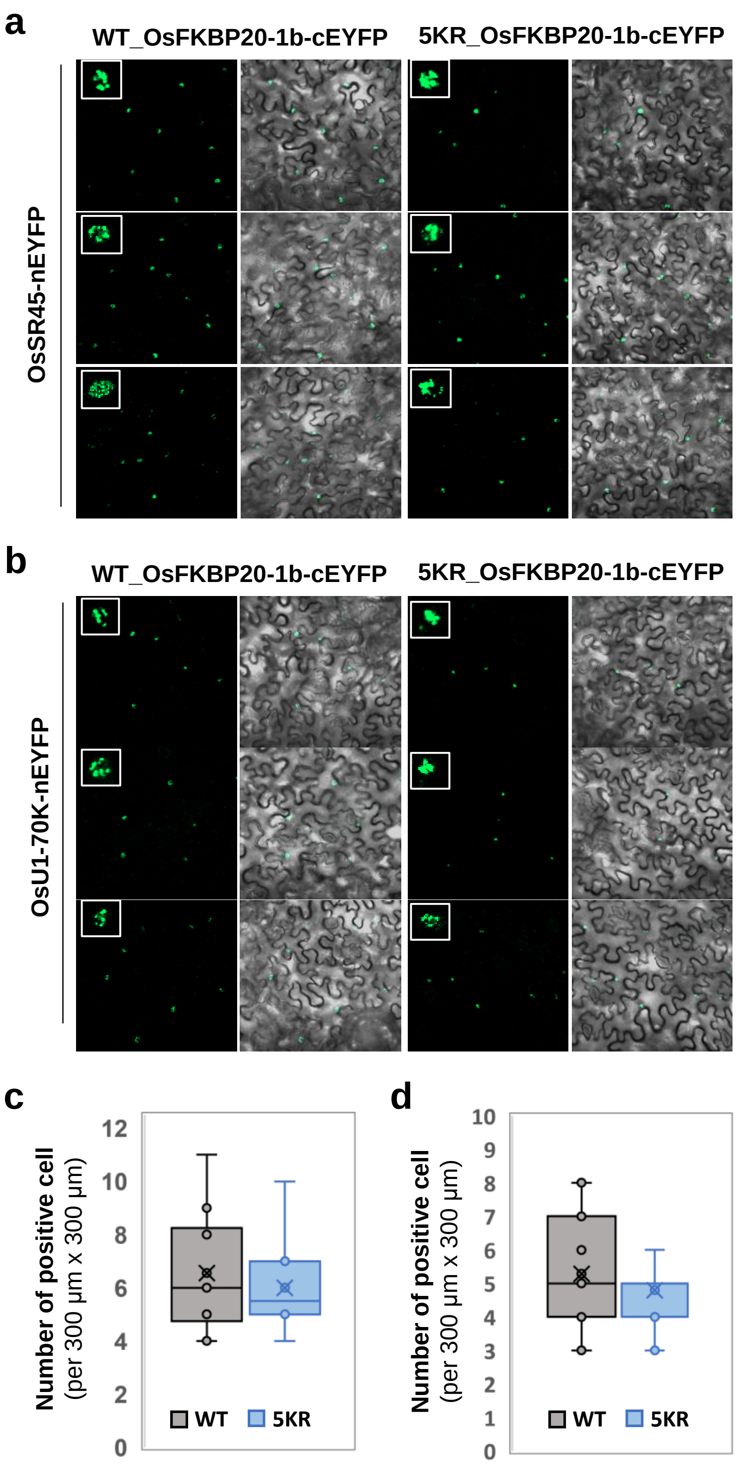

Supplementary Figure S4. Interaction between OsSR45 or OsU1-70K with OsFKBP20-1b in plant cells.

OsSR45 or OsU1-70k were fused to nEYFP and WT_ or 5KR_OsFKBP20-1b were fused to cEYFP for BiFC assay. Each construct was co-infiltrated into the leaves of *Nicotiana benthamiana* using agrobacterium. Two days after infiltration, the leaves were observed using a confocal microscope. (bar = 50 μm) **a and c**. Interaction of OsSR45 with WT_ or 5KR_OsFKBP20-1b. b **and d.** Interaction of OsU1-70k with WT_ or 5KR_OsFKBP20-1b. Number of GFP positive cells (in 300 x 300 um^2) of WT_ or 5KR_OsFKBP20-1b-GFP were counted (n = 7).

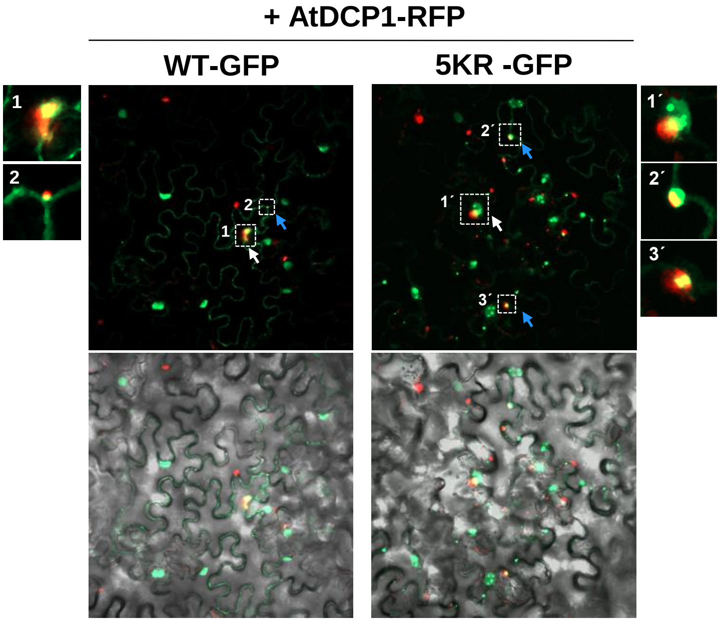

Supplementary Figure S5. Co-localization of OsFKBP20-1b with p-body.
GFP fused WT_ and 5KR_OsFKBP20-1b constructs and p-body marker protein, AtDCP1-RFP were transiently co-expressed in leaves of 4-week-old *Nicotiana benthamiana* using agro-infiltration. Two days after infiltration, plants epidermal cells were observed GFP and RFP fluorescence using confocal microscopy. The white square box 1 shows the enlarged nuclear area, and 2-3 shows the enlarged cytoplasmic focal area.

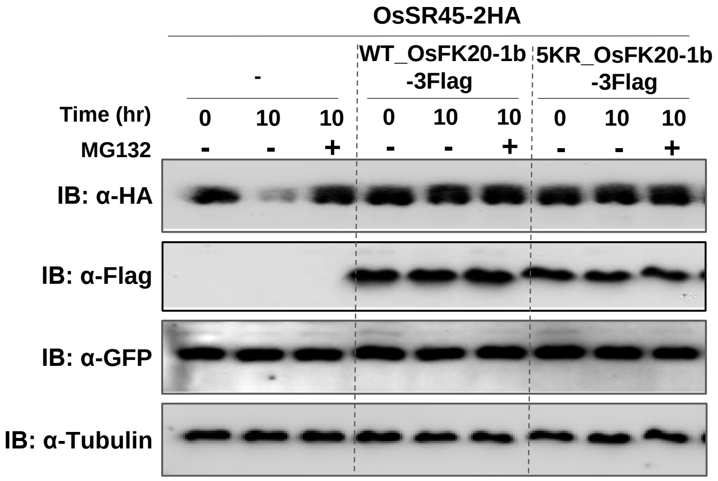

Supplementary Figure S6. OsSR45 protein stability under OsFKBP20-1b WT or 5KR co-expressed conditions.

Protoplasts were isolated from the shoots of 10-day-old *osfkbp20-1b k/o* seedlings and HA-tagged-OsSR45, flag-tagged-OsFKBP20-1b WT or 5KR and GFP were transiently expressed by PEG mediated transfection. Total proteins were isolated at 22 h (0 h) after protoplast transfection and incubated with or without MG132 for 10 h. The immunoblot assay was performed using HA, Flag, GFP and tubulin antibodies. Tubulin was used as a control to evaluate potential variation in the total amounts of protein loaded. GFP was used as a control for variation of transfection efficiency.

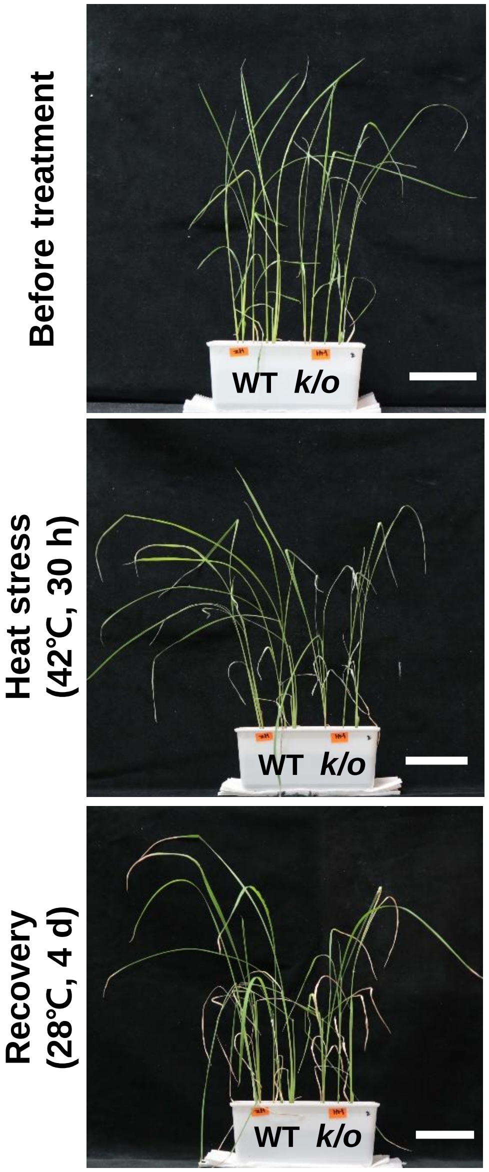

Supplementary Figure S7. Hypersensitive phenotype of *osfkbp20-1b* knock out plants under heat stress condition.

Wild type (WT, ZH11) and *osfkbp20-1b* k/o rice plants were grown on growth chamber (28℃, 16h light/ 8h dark, 50% humidity condition) until four leaf stage. Heat stress was treated at 42℃ for 30 hours and recovery performed at 28℃ for 4 days. Bar = 6 cm

Table S1. Gene-specific primers used in this study

Gene name	Primer Sequences F, forward [5'-3']	R, reverse [5'-3']	Applications
OsFkbp20-1b	GAATTCATGTCTGAGACCATAGATTTAACCGGAG	GGATCCCTATTTGGCCTTCTTTCCCTTGCCCTT	RT-PCR
**OsFKBP20-1b**	GACAGATCTCATGTCTGAGACCATAGATTTAAC	GACACTAGTTTTGGCCTTCTTTCCCTTGCCC	Localization
**OsFKBP20-1b**	TACCATGGGATGTCTGAGACCATAGA	AACCTCGAGTCAAGCGTAATCTGGAACATCGTATGGGTAAGCGTAATCTGGAACATCGTATGGGTATTTGGCCTTCTTTCCCTTGCC	In vitro SUMOylation
**OsFKBP20-1b**	TCTAGAATGTCTGAGACCATAGATTTAAC	CTCGAGTTTGGCCTTCTTTCCCTTGCCCT	BiFC
**OsSCE1**	ACTAGTATGTCGGGAGGGATCGCACGCGG	CTCGAGGGCGTGTTCGTGTTCAGGCCAAG	BiFC
**OsSCE2**	ACTAGTATGTCGGGGGGAATCGCGCGCGG	CTCGAGGACAATCGGAGGATACTGCTTGG	BiFC
**OsSCE3**	ACTAGTATGGCATCCGGAGGAGGCATCGC	CTCGAGCAGAGCTGAAGGATACTTCTTAG	BiFC
**OsSUMO1**	TCTAGAATGTCGTCGCCCGCGGGGGAGGA	ACTAGTGGCAGGCAGAGAGCCCCCAGTCT	BiFC
**OsSUMO2**	TCTAGAATGTCGGCCGCCGGGGAGGAGGA	ACTAGTGGCAGGCAGGCAGCCTCCAGTCT	BiFC
**OsSUMO3**	ACTAGTAGCGGCGCCACCGATCAGCTCCT	AGATCTGATGTTCGGCCGGTCTGGCATCA	BiFC
**OsSR45**	AGAACTAGTATGGCGAAGCCGCGCCGCGGC	AGAACTAGTCCTGCGCCTCGGGGAAGGTG	BiFC
**OsU1-70k**	AGA ACTAGT ATGGGCGACTACGGCAGCGGGAT	AGA ACTAGT GTTATTGTGCTCATCAGCAGCTTG	BiFC
**AtDCP1**	ACTAGATCTAATGTCTCAAAACGGGAAGAT	CGTAGATCTACTTGTTGAAGTGCATTTTGTAA	Localization
**AtUBP1b**	ACTAGATCTAATGCAGAGGTTGAAGC	ACGAGATCTCCCTGGTAGTACATGAGCTGC	Localization
OsLea3	TTGATCACTTGATTGTTCTTGGT	TGCCGGCCTCGTCTTCGGTCAT	qPCR
OsNac5	AAGGCCTCAGATCGGAGATTGAATG	GTAGATTCGGCACAACACCCAAT	qPCR
OsHsp90	ATGGCCTCGGAGACGGAGACGTT	CTCCATAAACTCCTTAGTCCCTG	qPCR
OsHsp101	CAAGGTGATCCTCTTCATCGACGAG	GCTCCACCTCCAGCTGAATCCTCTTC	qPCR
***OsLea3* AS**	ACCAGGACCAGGCTAGCTACC	TGCCGGCCTCGTCTTCGGTCAT	RT-PCR
OsHsfA2d	TCCTCCCACGCTTCTTCAAGCACAAC	CAAGTGCCTGCTGAGATCCAGGGAGAT	RT-PCR
OsHsfA2a	GTCCTGGTTTCGACGAGCTCCAT	TCGTCCACGACCTCGTACGTCTTGC	RT-PCR
OsAct1	CATGCTATCCCTCGTCTCGACCT	CGCACTTCATGATGGAGTTGTAT	RT-PCR, qPCR
